# Advancing Early Detection of Alzheimer’s Disease in the Primary Care Setting in the United States

**DOI:** 10.1002/alz.086582

**Published:** 2025-01-09

**Authors:** Michelle M. Mielke, H. Ricky Kurzman, Yan Helen Hu, Min Cho, Jonathan Liss, Jeffrey M Burns, Thomas O Obisesan, Michael Hornbecker, Arnold Pallay, Steven R. Smith, Lawrence S. Honig, Monica W Parker, Joanne Bell, Harald Hampel

**Affiliations:** ^1^ Wake Forest University School of Medicine, Winston‐Salem, NC USA; ^2^ Eisai, Nutley, NJ USA; ^3^ Eisai Inc, Nutley, NJ USA; ^4^ Columbus Memory Center, Columbus, GA USA; ^5^ University of Kansas Alzheimer's Disease Research Center, Fairway, KS USA; ^6^ Howard University College of Medicine, Washington, DC USA; ^7^ Witham Health Services, Lebanon, IN USA; ^8^ Atlantic Health System, Montville, NJ USA; ^9^ AdventHealth, Orlando, FL USA; ^10^ Columbia University Irving Medical Center, New York, NY USA; ^11^ Emory University Goizueta Alzheimer's Disease Research Center, Atlanta, GA USA; ^12^ Eisai Inc., Nutley, NJ USA

## Abstract

**Background:**

Recent advances in diagnostics have made it possible to identify early signs of the pathophysiological changes underlying Alzheimer’s Disease (AD) via blood tests. However, the use of blood‐based biomarkers (BBBMs) for the early detection of AD may be limited in primary care settings despite its potential for wide access and early detection of AD (PMID: 37295421)^.^ Therefore, there is a need to understand the barriers and facilitators of BBBM testing for AD in primary care.

**Method:**

We employed a combination of qualitative research, advisory board, and quantitative survey to engage with clinical/scientific advisors and community‐based physicians in primary care. In‐depth interviews with primary care physicians (PCPs) (n = 23) were conducted to understand the current awareness and clinical practice of AD identification in the primary care setting, followed by an advisory board to establish hypotheses about the barriers and opportunities of BBBM testing in the primary care setting. We subsequently conducted a quantitative survey with US PCPs (n = 600) to understand their attitudes and beliefs on screening and diagnosing AD in their practice, including opinions and willingness to use BBBM as part of the evaluation of individuals with mild cognitive impairment (MCI) or mild dementia.

**Results:**

Preliminary findings revealed that the majority of PCPs believe that MCI/mild AD is underdiagnosed and BBBM will likely facilitate better and faster diagnosis. Identified obstacles encompass inadequate financial coverage for BBBM testing, limited awareness among PCPs regarding the tests’ availability and value, limited awareness about monoclonal antibody (mAb) treatment for early‐stage AD, and lack of understanding of where and how to incorporate BBBM testing into primary care. Suggested solutions included education to equip PCPs with necessary knowledge on BBBM testing, clear guidelines on incorporating BBBMs simultaneously with clinical and cognitive assessments, and adequate financial coverage for both BBBMs and mAb treatments. Final survey results will be presented at the AAIC.

**Conclusion:**

The findings suggest that PCPs recognize the substantial value of BBBMs in detecting early AD, and that there is an urgent need for a concerted effort to improve awareness and address the barriers to adopting BBBMs in primary care.

